# Targeting Phosphatidylinositide3-Kinase/Akt pathway by BKM120 for radiosensitization in hepatocellular carcinoma

**DOI:** 10.18632/oncotarget.1978

**Published:** 2014-05-16

**Authors:** Wei-Lin Liu, Ming Gao, Kai-Yuan Tzen, Chiao-Ling Tsai, Feng-Ming Hsu, Ann-Lii Cheng, Jason Chia-Hsien Cheng

**Affiliations:** ^1^ Graduate Institute of Oncology, National Taiwan University College of Medicine, Taipei, Taiwan; ^2^ Graduate Institute of Clinical Medicine, National Taiwan University College of Medicine, Taipei, Taiwan; ^3^ Cancer Research Center, National Taiwan University College of Medicine, Taipei, Taiwan; ^4^ Department of Oncology, National Taiwan University Hospital, Taipei, Taiwan; ^5^ Department of Nuclear Medicine, National Taiwan University Hospital, Taipei, Taiwan; ^6^ Department of Internal Medicine, National Taiwan University Hospital, Taipei, Taiwan; ^7^ Molecular Imaging Center, National Taiwan University, Taipei, Taiwan

**Keywords:** Hepatocellular carcinoma, irradiation, phosphatidylinositol 3-kinase, radiation sensitivity, synergism

## Abstract

Tumor control of hepatocellular carcinoma by radiotherapy remains unsatisfactory. The phosphatidylinositol 3-kinase (PI3K)/Akt pathway plays a critical role in inhibiting cancer cell death. Elevated PI3K/Akt activity is associated with increased cellular resistance to irradiation. Our aim was to determine whether the inhibition of PI3K/Akt activity by a PI3K inhibitor, BKM120, contributes to the increased sensitivity of liver cancer cells to irradiation. The hepatocellular carcinoma cell lines (Huh7 and BNL) were used to evaluate the in vitro synergism between BKM120 and irradiation. Balb/c mice bearing ectopic BNL xenografts were treated with BKM120 and/or radiotherapy to assess the in vivo response. BKM120 increased cell killing by radiation, increased the expression of apoptotic markers, and suppressed the repair of radiation-induced DNA double-strand breaks. BKM120 pretreatment inhibited radiation-induced Akt phosphorylation and enhanced the tumor-suppressive effect and radiation-induced tumor cell apoptosis in ectopic xenografts. Inhibition of mTOR phosphorylation by rapamycin enhanced the radiosensitivity of BKM120-treated hepatocellular carcinoma cells. The synergism between BKM120 and irradiation likely inhibits the activation of Akt by radiation, leading to increased cell apoptosis and suppression of DNA-double-strand breaks repair in hepatocellular carcinoma cells. These data suggest that the BKM120/radiation combination may be a strategy worthy of clinical trials.

## INTRODUCTION

Hepatocellular carcinoma (HCC) is one of the most prevalent malignancies worldwide [1, 2]. Radiotherapy (RT) is being integrated with other treatments into multimodality treatments of HCC, especially for localized hepatic tumors refractory to conventional therapy [3, 4]. However, radiotherapeutic effects are typically unsatisfactory because of compromised liver reserve that makes optimal doses difficult to achieve [5]. Thus, strategies to enhance the therapeutic effects of radiation on hepatic tumors could yield clinical benefits for HCC patients.

A reasonable way to enhance the response of tumors to RT is by concomitantly using agents that inhibit radiation-activated signaling pathways. The phosphatidylinositol 3-kinase (PI3K) cascade is a critical component of survival signaling. PI3K-activated Akt (phosphorylated Akt) inhibits cell death pathways by inactivating pro-apoptotic proteins such as Bad and procaspase-9 [6]. Increased phosphorylation of Akt has been linked to decreased radiation responsiveness in various malignancies, including head and neck squamous cell carcinoma, lung carcinoma, glioblastoma, prostate adenocarcinoma, and breast cancer [7-12]. Thus, the inhibition of the PI3K signaling pathway may provide a directed approach supplemental to RT-induced cancer cell damage.

Several PI3K pathway inhibitors have been under development, including pure PI3K inhibitors (BKM120), compounds blocking both PI3K and mTOR (BEZ235), pure catalytic mammalian target of rapamycin (mTOR) inhibitors (RAD001), and the inhibitors of Akt. However, it remains unclear which type of inhibitor would be more effective clinically. Both the novel inhibitors, BKM120 and BEZ235, specifically inhibit the PI3K/Akt pathway in cancer cells with different mutant forms of the 110α catalytic subunit of PI3K [13]. BKM120 has been shown to induce the significant inhibition of cell proliferation and apoptosis in a variety of cancer cell lines, and is currently being investigated as a single agent in a phase I clinical trial of patients with solid tumors [14].

In this study, we demonstrated the suppression of radiation-activated PI3K/Akt by BKM120, leading to the enhanced apoptosis and DNA damage of human and murine HCC cell lines (Huh7 and BNL). Furthermore, our findings showed that BKM120 synergized with radiation to inhibit BNL cell survival and the growth of ectopic xenografts. The compensatory role of mTOR complex-related proteins in Akt activation was also involved with the effect of BKM120 and radiation.

## RESULTS

### Radiosensitization of hepatocellular carcinoma (HCC) cells by BKM120

**Figure 1:**
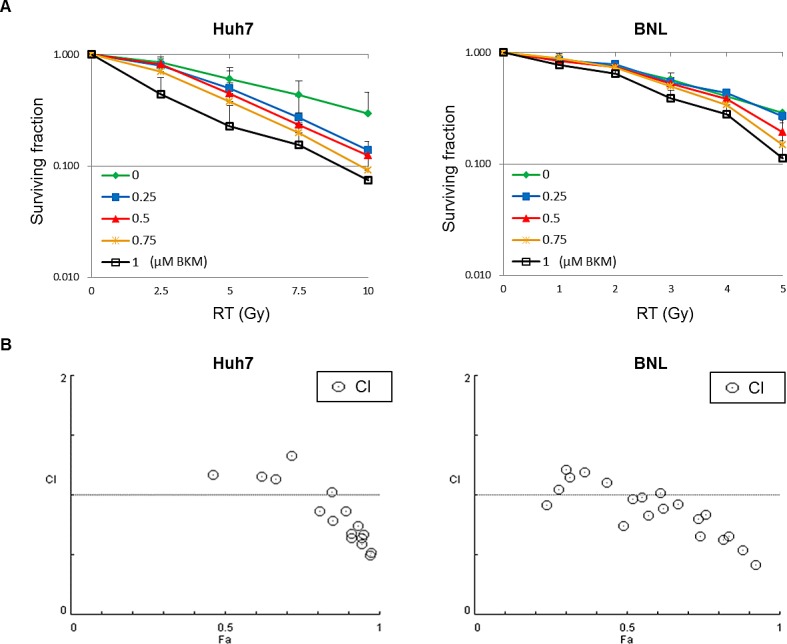
A PI3K inhibitor, BKM120, enhances the radiosensitization of hepatocellular carcinoma cell lines (Huh7 and BNL) (A) Quantitative results of clonogenic assays after combination treatment with BKM120 and irradiation. The images (magnified 100X) were used to count colonies containing more than 50 cells. At each dose level, the colony count is expressed as a fraction of the number in the corresponding control group. Lines, mean (n=3); Bars, S.D. (B) Combination indices (CI) are plotted against cell fraction affected (Fa). CI values <1 indicate synergism.

The activation of the PI3K/Akt pathway is associated with radioresistance. We investigated whether the PI3K inhibitor, BKM120, can enhance the radiosensitization of HCC cell lines (Huh7 and BNL). Cells were cultured at a density of 500 cells per well in six-well plates and pretreated with different doses of BKM120 (0.25–1 μM) for 1 hour and then irradiated with different doses. After 7 days, the cells were fixed, stained, and photographed (100X). Clonogenic cell survival decreased dose-dependently either with radiation (Huh7 : 1-10 Gy; BNL cells : 1–5 Gy) or BKM120 treatment (0.25–1 μM) (Fig. 1A). To determine if the interaction between BKM120 and irradiation was synergistic, combination indexes were calculated from the dose-response data. In Huh7 and BNL cells treated with higher doses of irradiation and BKM120, CI values of <1 were achieved and indicative of synergism (Fig. 1B).

### BKM120 combined with irradiation enhanced the apoptosis of Huh7 and BNL cells

**Figure 2:**
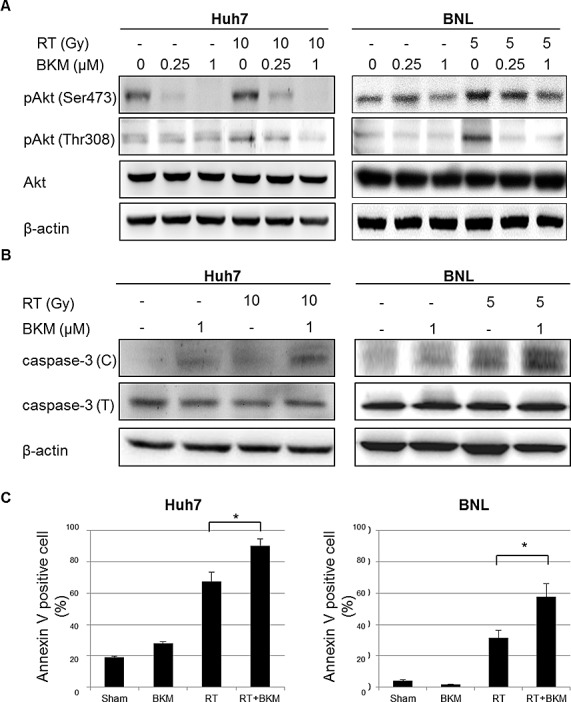
BKM120 inhibits radiation-activated PI3K/Akt signaling and enhances radiation-induced apoptosis in Huh7 and BNL cells (A) Western blotting shows that levels of phospho-Akt increase after irradiation and are inhibited by BKM120. (B) Western blots show the levels of the apoptotic markers caspase-3 (C: cleavage form; T: total form). (C) Early apoptotic Huh7 and BNL cells are identified using labeled with annexin V-FITC. The percentage of annexin V-positive cells in Huh7 and BNL cell cultures was calculated at 1 day.

Since radiation activates the PI3K/Akt pathway, we investigated whether BKM120 can suppress the radiation-activated phosphorylation of Akt. HCC cells were treated with irradiation and cell lysates prepared for Western blotting to detect phosphorylated Akt. Western blotting assays showed that the levels of phospho-Akt increased after irradiation (Fig. 2A). The increased phosphorylation of Akt by irradiation was inhibited by BKM120 (0.25 and 1μM) at 24 hours after irradiation (Fig. 2A). HCC cells were pretreated with BKM120 (1 μM) for 1 hour and then with radiation (Huh7 cells: 10 Gy; BNL cells: 5 Gy). After 24 hours, cell lysates were prepared for Western blotting to detect the apoptotic markers caspase-3 (C: cleavage form). Western blot analysis of cleaved caspase-3 revealed that pretreatment with BKM120 increased the expression of this apoptotic marker in response to irradiation (Fig. 2B). Subsequently, the cells were stained with FITC-labeled annexin V to identify apoptotic cells (Fig. 2C). Annexin V serves as a marker for the loss of plasma membrane asymmetry representing an early feature of apoptosis. As shown in Fig. 2C, the percentage of early apoptotic BKM120-treated cells increased significantly at 1 day after radiation when compared with sham-irradiated and irradiated-alone cells.

### BKM120 combined with irradiation enhanced DNA damage of Huh7 and BNL cells

**Figure 3:**
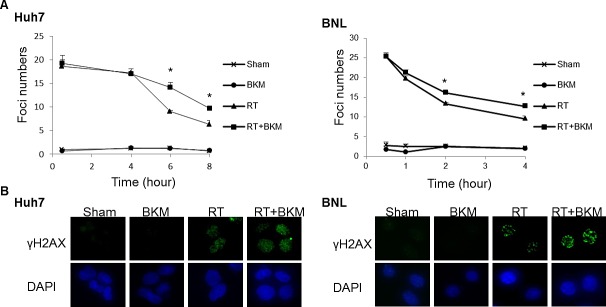
Inhibition of PI3K signaling by BKM120 leads to the persistence of DNA damage (A) The number of γ-H2AX foci/cell was counted in a minimum of 200 cells per treatment group. The average number of γ-H2AX foci/cell is shown. Error bars indicate S.D.*, P < 0.05. (B) The representative images of Huh7 cells from the 6-hour groups and BNL cells from 4-hour groups are shown.

We subsequently investigated whether PI3K inhibition modulates unrepaired DNA damage, via the assessment of phosphorylated histone H2AX (γ-H2AX), which forms foci at double-stranded DNA breaks and recruits DSB repair proteins. Correlating with the amount of unrepaired damage [15, 16], the number of γ-H2AX foci was quantified at different time points after irradiation (Fig. 3A). Huh7 and BNL cells on coverslips were exposed to vehicle only (DMSO) or BKM120 (1 μM) for 1 hour, followed by irradiation (RT) with 10 Gy for Huh7 cells and 5 Gy for BNL cells. Replicated groups of cells were fixed either immediately after RT (30 mins) or the indicated hours after RT. All cells were then stained for γ-H2AX or nuclei (DAPI). Radiation-induced γ-H2AX foci detected via immunofluorescence were apparent within 30 minutes of irradiation and substantially decreased in number by 8 hours in Huh7 cells and 4 hours in BNL cells after radiation alone. In contrast, the addition of BKM120 to Huh7 and BNL cells led to retention of these foci in a substantial percentage of cells 6 hours in Huh7 cells and 4 hours in BNL cells after irradiation, indicating unrepaired DNA damage. Fig. 3B shows the immunofluorescence staining of γ-H2AX of Huh7 cells at 6 hours and BNL cells at 4 hours after radiation.

### The combination of BKM120 and radiotherapy enhanced growth inhibition of ectopic BNL xenograft tumors

**Figure 4:**
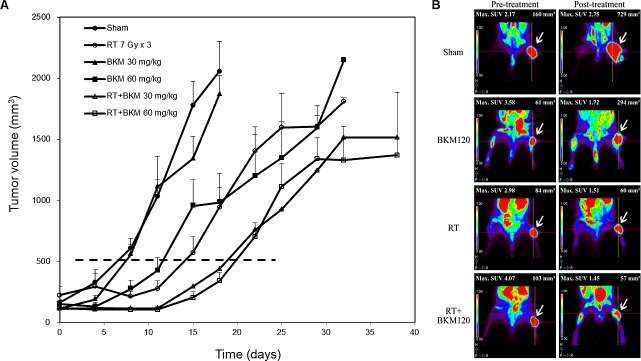
Combined BKM120 and radiotherapy (RT) enhances tumor suppressive activity in two BNL xenograft models (A) Balb/c mice bearing subcutaneous BNL tumors. Points, mean; bars, S.D. (B) Mice bearing ectopic BNL xenografts. Positron emission tomography/computed tomography was performed on day 8. Representative images are shown. Arrows indicate the viable right thigh tumors. The standard uptake value (SUV) and the viable volume of tumor are shown above the image.

**Figure 5:**
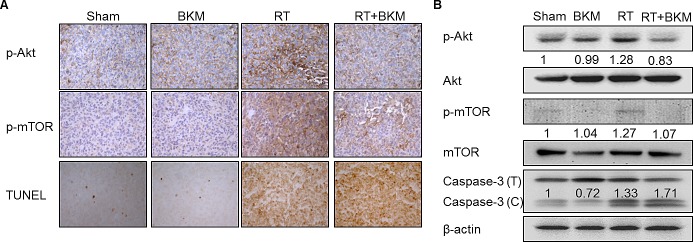
Combined radiotherapy (RT) and BKM120 inhibits RT-activated PI3K/Akt signaling and enhances BNL cell apoptosis *in vivo*. Mice bearing ectopic tumors were sacrificed on day 8 (A) Microscopic images (200X) are shown of immunohistochemically stained tumor tissue sections with phospho-Akt, phosphor-mTOR, and apoptosis marker. (B) Western blots assays of phospho-Akt, phosphor-mTOR, and caspase-3 are shown for a representative mouse in each group. Combined radiotherapy (RT) and BKM120 inhibits RT-activated PI3K/Akt signaling and enhances BNL cell apoptosis *in vivo*. Mice bearing ectopic tumors were sacrificed on day 8. Microscopic images (200X) are shown of immunohistochemically stained tumor tissue sections. Western blots assays of (A) phospho-Akt, (B) phospho-mTOR, and (C) apoptosis markers are shown for a representative mouse in each group.

Balb/c mice bearing subcutaneous BNL tumors were randomized into 4 groups (n = 5 in each group) to receive RT alone (7 Gy per day on day 3–5), oral BKM120 (30 mg/kg/day and 60 mg/kg/day from day 1–7) alone, combined BKM120 and RT, or sham treatment. Daily oral treatment with BKM120 (30 mg/kg and 60 mg/kg for 7 days) in combination with RT on day 3–5 suppressed the growth of xenograft tumors to a greater extent than RT alone (Fig. 4A). BKM120 itself did not satisfactorily control tumor growth. The addition of BKM120 enhanced RT-induced suppression of BNL tumor growth. The number of days until the tumor volume reached 500 mm3 in the RT+BKM120 (30 mg/kg and 60 mg/kg) groups and RT alone group were 19, 20 days and 14 days, respectively. Mice bearing ectopic BNL xenografts received BKM120 (30 mg/kg/day) alone, RT alone, combined RT and BKM120, or sham treatment. One day after the treatment (day 8), 18F-FDG micro-PET/CT images showed that tumor viability was much decreased after combined BKM120 and RT, as compared to either modality alone or sham treatment (Fig. 4B). Metabolic tumor volume was not reduced by treatment with BKM120 alone but partially reduced by RT alone. Importantly, co-treatment with BKM120 at 30 mg/kg and RT significantly improved this radiotherapeutic effect. The phosphorylation of Akt and mTOR was assessed by Western blotting and immunohistochemical assays in BNL tumors harvested at 8 days after initiation of treatment. Combined treatment with RT and BKM120 suppressed levels of radiation-activated Akt and mTOR in tumor tissues (Fig. 5A and 5B). In addition, immunohistochemical and Western blot evaluations of apoptosis (TUNEL and caspase-3) revealed that the combination treatment induced apoptosis in xenograft tumors (Fig. 5A and 5B).

Taken together, the results indicate that BKM120 radiosensitizes BNL cells in vitro and in vivo, and suggest that radiosensitization occurs through the inhibition of the radiation-activated PI3K/Akt pathway.

### Inhibition of mammalian target of rapamycin (mTOR) phosophorylation by rapamycin further enhanced radiosensitization of BNL cells with BKM120

**Figure 6:**
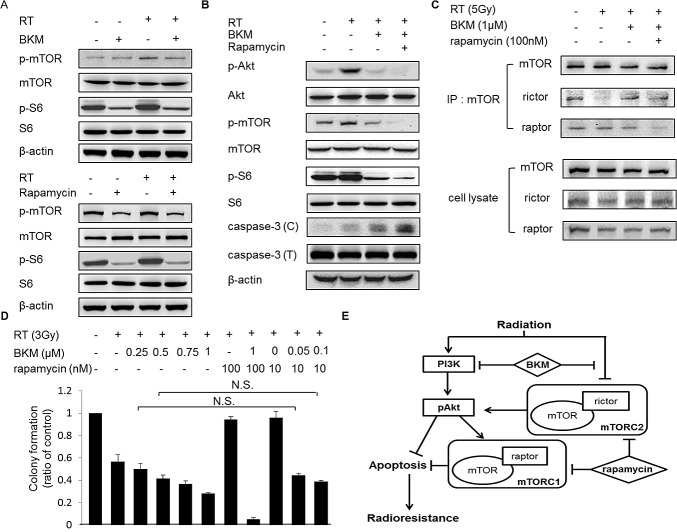
The addition of rapamycin to BKM120 enhances the inhibition of mTOR and Akt phosphorylation and increases caspase-3 activation in irradiated BNL cells (A) Western blot shows level of phospho-mTOR and phospho-S6 kinase in BNL cells pretreated with BKM120 (1 μM) or rapamycin (100 nM) for 1 hour and then irradiated (RT; 5 Gy). (B) Western blot shows levels of phospho-Akt, phospho-mTOR, phospho-S6 kinase and caspase-3 in BNL cells pretreated with BKM120 (1 μM) and rapamycin (100 nM) for 1 hour and then irradiated (RT; 5 Gy). (C) The effect of BKM120 and rapamycin on the integrity of mTORC1 and mTORC2. (D) Quantitative results of clonogenic assays after the combination treatment with BKM120, rapamycin, and RT. N.S.: not significant. (E) Effects of the PI3K inhibitor BKM and the mTOR inhibitor rapamycin on the PI3K signal transduction pathway in HCC cells with radiation treatment.

mTOR is a downstream effector in the PI3K/Akt pathway, which regulates protein synthesis, metabolism, growth, cell cycle progression, and survival. In mammalian cells, two distinct mTOR-containing complexes have been identified: rapamycin-sensitive mTOR complex (mTORC1; containing raptor) and rapamycin-insensitive complex (mTORC2; containing rictor). mTORC2 phosphorylates Akt at S473, a key regulator of cell survival. BKM120 did not totally inhibit the phosphorylation of mTOR and phospho-S6 kinase after radiation (Fig. 6A). The treatment of rapamycin partially reduced the levels of mTOR and phospho-S6 kinase (Fig. 6A). Notably, combined BKM120 and rapamycin suppressed the radiation-induced Akt and the phosphorylations of its downstream signaling components mTOR and S6 kinase more than BKM120 alone, and further increased caspase-3 activation (Fig. 6B). Irradiated BNL cells pretreated with BKM120 (1 μM) and/or rapamycin (100 nM) were lysed in RIPA lysis buffer, and 500 μg of each lysate was subjected to immunoprecipitation with the mTOR antibody. The amounts of mTOR, rictor, and raptor in mTOR immunoprecipitates were analyzed on immunoblots. By immunoprecipitation, radiation alone suppressed the forms of mTORC2 while BKM120 induced the binding of mTOR and rictor in irradiated BNL cells. Rapamycin inhibited the assembly of mTOR/raptor and mTOR/rictor more than BKM120 alone (Fig. 6C). Thus, we show that inhibition of the assembly of mTOR/rictor by rapamycin may enhance the radiosensitization of BNL cells by BKM120. By combining rapamycin (10 nM) with low-dose BKM120 (50 nM and 100 nM) and irradiation, the clonogenic survivals were similar to those by higher-dose BKM120 (250 nM and 500 nM) and irradiation (Fig. 6D). It is likely that the combined inhibition of PI3K/mTOR pathways with irradiation will have synergistic effects at the level of apoptosis, via effects upon mTOR complexes formation, as well as through increased activation of Akt (Fig. 6E).

## DISCUSSION

HCC is one of the most lethal cancers worldwide, and few curative options are available to patients with advanced disease. The increasing use and improved technology of RT have gradually established it as an alternative treatment regimen [17]. Partial regression is the most common sublethal response of HCC to RT [3]. To increase safety and reduce side effects of RT, pharmacological radiosensitization has been increased by using it in combination with medicinal compounds.

Unregulated signaling through the PI3K pathway is linked to the development of certain types of cancer including HCC [6, 18, 19]. In recent clinical study reports, activation of PI3K/Akt/mTOR and MAPK/ERK pathways is correlated with tumor progression and worse survival of patients [20, 21]. The frequent overexpression of the PI3K/Akt/mTOR pathway in HCC is believed to contribute to its aggressive phenotype [22]. Recent study also showed that co-targeting mTOR and MEK may be effective in patients with malignant peripheral nerve sheath tumors [23]. Uncontrolled signaling through the PI3K pathway may be a critical target for therapeutic intervention. Thus, the inhibitors of the PI3K/Akt/mTOR pathway would have potentially significant anticancer effects. Many of the PI3K inhibitors currently in pre-clinical or clinical development inhibit all of the catalytic subunit isoforms of class IA PI3Ks [24, 25]. BKM120 is a newly developed oral pan-class I PI3K inhibitor which selectively inhibits PI3Kα, β, γ, and δ and mutated PI3K. BKM120 has been shown to inhibit the phosphorylation of Akt and to induce cell death in several cancer cell lines [26, 27]. It had been studied and had promising results in patients with advanced solid tumor in a phase I study [14]. Our pre-clinical study similarly demonstrated that treatment with BKM120 enhanced the effect of RT by inhibiting RT-induced Akt phosphorylation of HCC cells.

RT-induced Akt activation is a common mediator of cell survival signals through multiple intracellular signaling pathways including PI3K [28, 29]. Several studies have shown that the activation of PI3K/Akt survival pathway is associated with radioresistance in many cancer cell lines [30-32]. Use of the corresponding PI3K inhibitor is a reasonable solution to this problem. Studies on PI3K inhibition in other human cancer cell lines have shown that the nonselective PI3K inhibitors, such as LY294002 and wortmannin, have radiosensitizing effects and induce cell apoptosis [33, 34]. In the present study, we explored the anticancer effects of BKM120 against RT-treated HCC cells. As expected, BKM120 dose-dependently inhibited the RT-induced phosphorylation of Akt, effectively suppressed the PI3K/Akt signaling activated by irradiation, and thereby induced apoptosis in RT-treated HCC cells.

The formation of γ-H2AX foci increases the local concentration of repair proteins and facilitates repair of DSBs [35]. Akt phosphorylation has been reported to mediate DNA damage repair, and treatment with an Akt inhibitor has been reported to suppress DNA-DSB repair through modulating the phosphorylation of the DNA-protein kinase catalytic subunit [36]. Here we showed the potential mechanism for radiosensitization by a PI3K inhibitor through the disruption of the DNA damage repair process. We found that down-regulation of PI3K/Akt signaling by the PI3K inhibitor BKM120 led to the persistence of unrepaired double strand DNA damage by irradiation. To our knowledge, this is the first report to clearly characterize BKM120 as a radiosensitizer and its mechanism in both *in vitro* and *in vivo* xenograft models.

The PDK1–Akt (Thr308) axis controls TSC2 phosphorylation, which activates mTORC1 and subsequently S6K1 with feedback mechanism on Akt phosphorylation [37]. It was reported that radiation induced Thr308 Akt phosphorylation in the livers of both p53 wild-type and knockout mice. However, radiation decreased phospho-S6 kinase in the livers of p53 wild-type mice, and had the negative effect of p53 on mTOR [38]. Radiation induced tumor suppressor p53 is involved in the complex response to radiation, including cell cycle regulation, DNA repair, and apoptosis [39, 40]. Inhibition of mTOR by p53 is partially involved in the suppressed cellular senescence, and converts it into quiescence [41-43]. Here we showed that a combination of the PI3K inhibitor BKM and rapamycin acts synergistically to induce cell death after radiation treatment (Figure 6B). The activation of p53 after the combination of irradiation and sensitizers may be important in the regulation of mTOR on Akt [44, 45]. Further investigations are warranted to determine the exact mechanism by which PI3K/Akt/mTOR and p53/mTOR signaling cascades become activated in radiation treated tumors.

BKM120 does not bind to the Ser774 residue of PI3K; such binding was proposed as important and effective in mTOR inhibition [46]. mTOR can affect PI3K/Akt signaling through the S6K-IRS1 feedback loop and the induction of Akt phosphorylation by mTORC2 [47, 48]. Such compensation limits the therapeutic effect of single-pathway mTOR inhibitors. Agents such as BEZ235 (Novartis, East Hanover, NJ) and EX147 (Exelixis, San Francisco, CA) are dual PI3K/mTOR inhibitors and thus may suppress the feedback loops. Rapamycin was originally thought to only inhibit mTORC1. However, it has been recently shown that the long-term rapamycin treatment also suppresses mTORC2 activity [49]. In this study, BKM120 slightly reduced the phosphorylation of mTOR at the Ser2448 residue, which regulates the binding of raptor and rictor to mTOR [50], and inhibitors of both PI3K and mTOR blocked Akt phosphorylation more completely, which resulted in improved radiation sensitization in HCC cells. The radiosensitizing effect of dual inhibition was better than that of PI3K inhibition alone. These data indicate that inhibition of both PI3K and mTOR might prevent PI3K feedback signaling and further enhance radiation-induced cell killing.

In conclusion, in the *in vitro* HCC cell models and *in vivo* ectopic tumor model, we demonstrated the radiosensitizing activity of BKM120, an orally bioavailable PI3K inhibitor. BKM120 mediates its effect on HCC cells by inhibiting radiation-activated PI3K/Akt signals, thereby causing enhanced cell apoptosis and DNA damage. The addition of mTOR inhibitor further increases the radiosensitivity of BKM120-treated HCC cells. The findings may have clinical implications for the development of novel therapeutic strategies for HCC.

## MATERIALS AND METHODS

### HCC cell lines

Human HCC cell line Huh7 was obtained from JCRB cell bank (Okayama, Japan) and the murine HCC cell line, BNL, was obtained from American Type Culture Collection (ATCC, Manassas, VA, USA). Cells were cultured in DMEM supplemented with 10% fetal bovine serum and 50 U/ml penicillin/streptomycin. Cells were cultured at 37°C in a humidified atmosphere of 5% CO2 and 95% air.

### Reagents

BKM120 was provided by Novartis (Basel, Switzerland). For in vitro studies, stock solutions of BKM120 were prepared in dimethyl sulfoxide (DMSO) and diluted in culture medium containing 10% fetal bovine serum. For in vivo studies, BKM120 was suspended in a vehicle (NMP/PEG300 [10:90, v/v]) for oral administration to Balb/c mice bearing xenograft tumors.

### Irradiation of cells

HCC cells in culture flasks were irradiated with different doses of radiation, using a Primus 6-MV photon linear accelerator (Siemens Oncology Medical Systems, Inc., Concord, CA, USA). The distance from the radiation source to the bottom of the flask was set at 100 cm.

### Colony formation assay

Cells (500/well) were seeded in six-well plates and treated with different doses of radiation (0 Gy–10 Gy for Huh7 cells and 0 Gy–5 Gy for BNL cells) following one-hour pretreatment with various doses of BKM120 (0.25–1 μM) or DMSO vehicle. Cells were then cultured for an additional 7 days, after which the number of colonies (clusters of more than 50 cells) was counted in each well using an inverted phase-contrast microscope at 100X magnification and photographed. The effect on colony number was analyzed using CompuSyn software (ComboSyn, Inc., Paramus, NJ, USA). The combination index (CI) was calculated by the Chou-Talalay equation (Chou TC, Talalay P. Trends Pharmacol Sci 1983;4:450-454). The general equation for the classic isobologram (CI=1) is given by: CI=(D)1/(Dx)1+(D)2/(Dx)2 (A) where (Dx)1 and (Dx)2 in the denominators are the doses (or concentrations) of D1 (drug #1) and D2 (drug #2) alone that gives x% inhibition, whereas (D)1 and (D)2 in the numerators are the doses of D1 and D2 in combination that also inhibits x% (isoeffective). The (Dx)1 and (Dx)2 can be readily calculated from the Median-effect equation: Dx=Dm [fa/1-fa)]1/m (B) where Dx is the median effect dose obtained from the anti-log of the X-intercept of the median-effect plot, X-log (D) versus, Y=log[fa/1-fa)], or Dm=10(Y-intercept)/m, fa is the fraction affected by dose D and m is the slope of the median-effect plot. From (Dm)1, and (Dx)2 and D1+D2, an isobologram can be constructed based on Eq. A: as CI<1 indicates synergism, CI=1 indicates an additive effect, and CI>1 indicates antagonism.

### Western blot analysis

The proteins (50 μg of protein/aliquot) in aliquots of cell lysates were separated by sodium dodecylsulfate polyacrylamide gel electrophoresis (SDS-PAGE; 8–15% polyacrylamide), transferred to polyvinylidene difluoride (PVDF) membranes, and immunoblotted with various antibodies. Bound antibodies were detected using appropriate peroxidase-coupled secondary antibodies followed by enhanced chemiluminescence (ECL, Boehringer Mannheim, Mannheim, Germany). Antibodies to phospho-Akt and phospho-mTOR were obtained from Cell Signaling, Inc. (Burlingame, CA, USA), phospho-Akt (Ser473 and Thr308) and phospho-mTOR (Ser2448), phosphor-p70 S6 kinase (Thr389) and caspase-3 from Cell Signaling Technology (Danvers, MA, USA), S6 and beta-actin from Santa Cruz Biotechnology (Santa Cruz, CA, USA), and phospho-H2AX (Ser139) from Millipore Corporation (Billerica, MA, USA). Beta-actin was used as the loading control.

### Determination of apoptosis with fluorescence microscopy

Apoptotic cells were detected using the annexin V/FITC apoptosis detection kit (AVK050, Strong Biotech, Taipei, Taiwan) according to the manufacturer's instructions. The annexin V-positive cells were examined using a Zeiss Axio Imager A1 fluorescence microscope.

### γH2AX immunofluorescence microscopy

Huh7 and BNL cells were plated on 4-well chamber slides, allowed to attach overnight, and exposed to ionizing irradiation of 10 Gy or 5 Gy either alone or combined with 1 μM of BKM120. After treatment, Huh7 cells were incubated for 30 minutes, 4, 6, and 8 hours, BNL cells were incubated for 30 minutes, 1, 2, and 4 hours, washed three times with ice-cold phosphate-buffered saline (PBS), fixed in 4% formaldehyde/PBS for 30 minutes, permeabilized in 0.5% Triton X-100 in PBS for 30 minutes, blocked in 5% bovine serum albumin for 1 hour at room temperature, incubated with the antibody (fluorescein isothiocyanate [FITC]-conjugated anti-phospho-histone γH2AX [Ser139; 1:500; Millipore, Billerica, MA, USA]) for 16 hours at 4.C in the dark, washed with PBS, and mounted in Vectashield mounting medium containing diamidino-2-phenylindole (Vector Laboratories, Burlingame, CA, USA). γ-H2AX foci were examined using a Zeiss Axio Imager A1 fluorescence microscope. In each sample, the number of γ-H2AX foci per nucleus was counted using an automated foci counter under high power field, and an average of 150 nuclei were analyzed. The average number of γ-H2AX foci per nucleus represents the amount of double strand breaks (DSB).

### Immunoprecipitation

Whole protein lysates containing 500 μg of protein were prepared using radioimmunoprecipitation assay (RIPA) lysis buffer and immunoprecipitated at room temperature for 2 h using the Catch and Release v2.0 Reversible Immunoprecipitation System (Millipore). mTOR was immunoprecipitated with primary antibody diluted 1:100 in a total volume of 500 μl. Protein complexes were eluted from the column using 70 μl of 1× denaturing elution buffer in accordance with the manufacturer's directions. About 30 μl of eluted proteins was separated by SDS-PAGE using a 8% resolving gel, transferred to a PVDF membrane, and probed overnight with primary antibodies to mTOR, rictor, and raptor.

### *In vivo* ectopic tumor model

Male Balb/c mice (6 weeks of age) were obtained from the National Laboratory Animal Center and used for ectopic (subcutaneous) xenograft implantation. Body weights were measured weekly. Ectopic tumors were established by subcutaneous injection of BNL cells (1 × 10^6^) into the right hind leg of mice. Tumor volumes were measured with a set of calipers and calculated using a standard formula: width^2^×length/2. All experimental procedures using these mice were performed in accordance with protocols approved by the National Taiwan University Institutional Animal Care and Use Committee.

As the tumors became established (mean starting tumor volume =147 mm^3^), the mice were randomized into 6 groups to receive the following treatments: (1) methylcellulose/Tween 80 vehicle; (2) BKM120 (30 mg/kg/day and 60 mg/kg/day of body weight) on day 1–7; (3) methylcellulose/Tween 80 vehicle plus 7 Gy/day of RT on day 3–5; (4) BKM120 (30 mg/kg/day and 60 mg/kg/day of body weight) plus RT (7 Gy/day). Small animal positron emission tomography/computed tomography (PET/CT) scans with [18F]-2-fluoro-2-deoxy-D-glucose (FDG) were performed one day before and on day 8 of treatment. The mice were intravenously injected with 14 MBq (378 Ci) of 18FDG in saline via the tail vein.

### Irradiation of mice

Mice were immobilized using a customized harness. With the body shielded, the thigh tumor was irradiated with a half-beam rectangular field. A 6-MV photon linear accelerator was used to irradiate the thigh tumor with 3 fractions of 7 Gy/day on day 3–5.

### Histological evaluation

Mice from each group were sacrificed on day 8. The tumor was fixed in 10% neutral buffered formalin and processed for histopathological and immunohistochemical staining as well as Western blot assay. After fixation, tumor tissues were embedded in paraffin blocks and sectioned (5 μm). Tumor cells were detected in representative stained sections. The expressions of phospho-Akt and phospho-mTOR were evaluated after immunohistochemical staining using specific antibodies. DNA fragmentation was assessed using the DeadEnd Colorimetric Apoptosis Detection System (Promega, Madison, WI, USA). Tissue sections were deparaffinized by immersing slides in fresh xylene, dehydrated in a graded series of ethanols, and subjected to the terminal deoxynucleotidyltransferase–mediated dUTP-biotin nick-end labeling (TUNEL) assay following instructions of the manufacturer. Nucleotide incorporation was detected by treatment with horseradish peroxidase–conjugated streptavidin and enzyme substrate.

### Statistical analysis

The tumor volume data satisfied the assumptions of normality and homogeneity of variance for the use of parametric analysis; thus, group means for the ectopic tumor models were compared with a one-way analysis of variance followed by Fisher's least significant difference method for multiple comparisons. Differences were considered significant at P < 0.05.
